# Berberine chloride suppresses pancreatic adenocarcinoma proliferation and growth by targeting inflammation-related genes: an in silico analysis with in vitro and vivo validation

**DOI:** 10.1007/s00280-024-04663-7

**Published:** 2024-03-19

**Authors:** Lin-jie Ruan, Ju-ying Jiao, Chienshan Cheng, Yuan Zhang, Zhang-qi Cao, Ba He, Zhen Chen

**Affiliations:** 1 Department of Integrative Oncology, Fudan University Shanghai Cancer Center, No. 270 Dongan Rd., Xuhui district, Shanghai, China; 2 Department of Oncology, Shanghai Medical College, Fudan University, Shanghai, China

**Keywords:** Molecular mechanism, Network pharmacology, Tumor microenvironment, Tumor suppression

## Abstract

**Purpose:**

Targeting inflammatory crosstalk between tumors and their microenvironment has emerged as a crucial method for suppressing pancreatic adenocarcinoma (PAAD) progression. Berberine (BBR) is a natural pentacyclic isoquinoline alkaloid known for its anti-inflammatory and antitumor pharmacological effects; however, the mechanism underlying PAAD suppression remains unclear. We aim to investigate the effects of BBR on PAAD progression and their underlying mechanisms.

**Methods:**

The prognostic value of inflammation-related genes in PAAD was assessed using bioinformatics analyses, then the pharmacological effects and potential mechanisms of BBR on PAAD will be investigated in silico, in vitro, and in vivo.

**Results:**

Fifty-eight prognostic inflammation-related genes were identified in PAAD, which were shown to have good sensitivity and specificity using a novel inflammation-related gene risk-prognosis prediction model. Among these, four candidate genes (*CAPS3, PTGS2, ICAM1*, and *CXCR4*) were predicted as targets of BBR in PAAD in silico. Molecular docking simulations showed that the four key targets docked well with BBR. Further BBR treatment suppressed cell proliferation, colony formation, and induced cell cycle arrest in vitro. Moreover, BBR exhibited a significant tumor-suppressive effect in murine subcutaneous xenografts without macroscopic hepatic and renal toxicities. In addition, BBR downregulated *CAPS3*, *PTGS2*, *ICAM1*, and *CXCR4* protein expression.

**Conclusion:**

This study not only elucidated the prognostic value of inflammation-related genes in PAAD but also demonstrated the potential of BBR to inhibit PAAD by targeting these genes.

**Supplementary Information:**

The online version contains supplementary material available at 10.1007/s00280-024-04663-7.

## Introduction

Pancreatic adenocarcinoma (PAAD) is a highly malignant tumor with a rising incidence, and despite numerous efforts to improve therapeutic outcomes, its prognosis remains poor, with a 5-year overall survival (OS) of 9% [1, 2]. Characterized by its unique inflammatory microenvironment with abundant cross-talk between tumor cells and immune cell-secreted cytokines [3], PAAD is a highly progressive form of cancer. It is therefore urgent to seek new therapeutic strategies with effective targets.

Exploration of natural products is crucial for discovering innovative therapeutic agents that possess desirable attributes, such as high accessibility, low cost, and minimal toxicity [4, 5]. Berberine (BBR) is a pentacyclic isoquinoline alkaloid (2,3-methylenedioxy-9,10-dimethoxy protoberberine chloride, C_20_H_18_NO_4_^+^) derived from diverse pharmacological plants such as *Coptis chinensis Franch*, *Phellodendri Chinensis Cortex*, and *Barberry Root*; it exhibits a wide-ranging therapeutic potential against various diseases, including diabetes, hypertension, depression, obesity, and inflammation, and is a promising candidate for cancer treatment [6]. In our previous study, BBR was shown to suppress PAAD growth [7]; however, the extent to which it regulates inflammation-related genes requires further investigation.

**Fig. 1:**
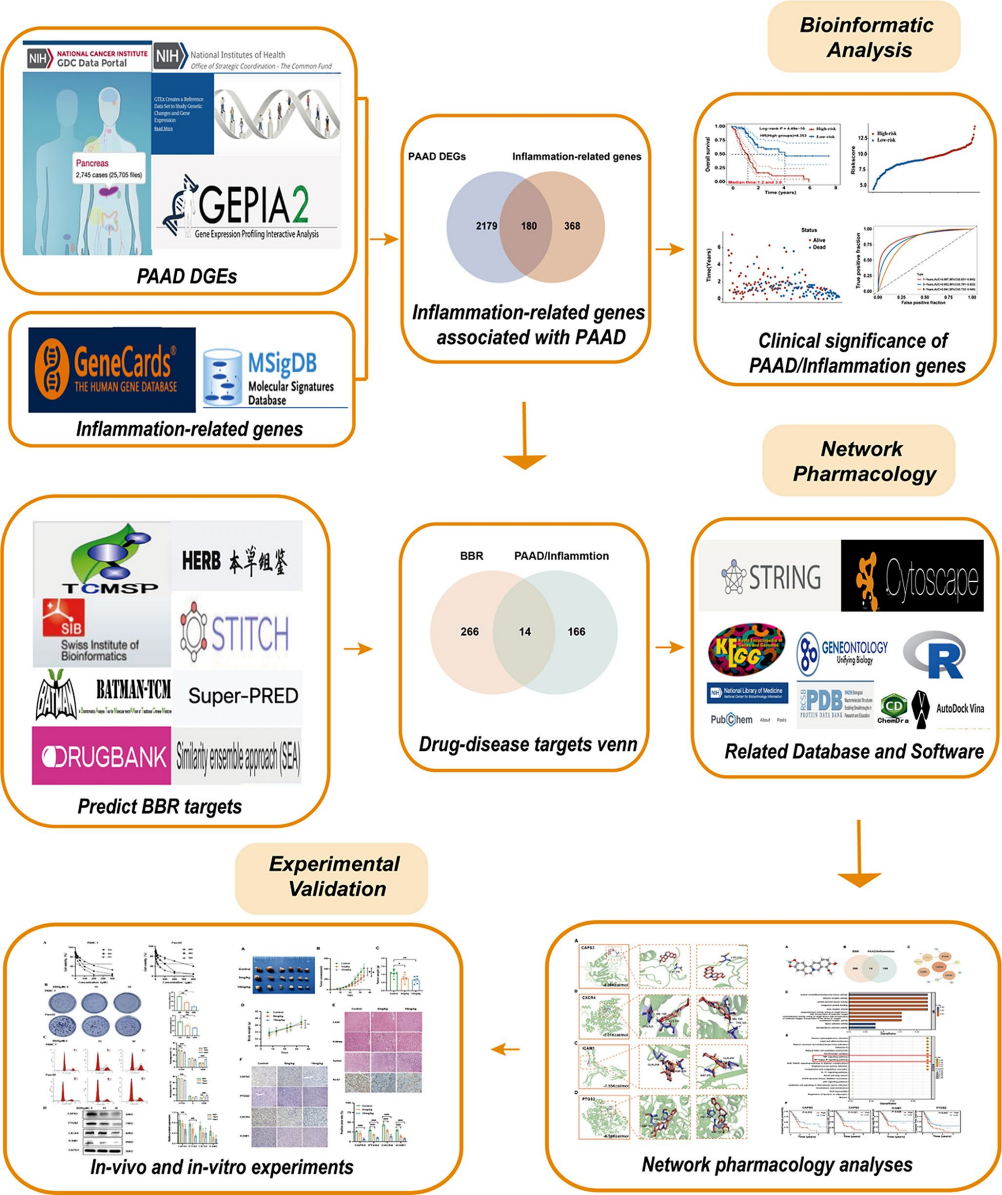
Study workflow

The relationship between inflammation and tumor progression is widely recognized. This study specifically examined inflammation-related genes in PAAD and assessed their impact on prognosis. Furthermore, the study investigated the therapeutic potential of these genes as targets and explored whether BBR, known for its anti-inflammatory and anti-tumor properties, could impede PAAD progression by targeting inflammation-related genes. The present study provides new insights and scientific evidence for the clinical application of BBR interventions for PAAD. A visible study workflow is provided in Fig. 1.

## Materials and methods

### Identification of inflammation-related genes in PAAD

The differentially expressed genes (DEGs) between PAAD tissues and adjacent non-tumor tissues was identified by the GEPIA2 web tool (http://gepia2.cancer-pku.cn//) with a false discovery rate of < 0.05, and a | log fold-change | of > 1 [8]. Subsequently, the gene symbols were converted on the UniProt website (https://www.UniProt.org/) [9], and the “protein coding” gene was extracted and filtered. Inflammation-related genes were mined from Genecards (https://www.genecards.org/) [10] and Molecular Signatures Databases (MSigDB, https://www.gsea-msigdb.org/) [11] and compared to identify overlapping genes in PAAD and inflammation. Volcano maps were used to demonstrate differential genes expression.

### Clinical significance analysis of inflammation-related genes in PAAD

Prognosis-associated genes (risk ratio = 1, *P* < 0.05) were screened by univariate Cox proportional risk regression analysis. These genes were then overlapped with the previously identified DEGs for subsequent analysis. A multigene signature with overlapping candidate genes was constructed by multivariate Cox regression analysis for predicting PAAD prognosis [12]. In order to compare the survival differences between the high- and low-risk groups, the Kaplan–Meier survival curves was performed by the log-rank test. The accuracy of the predictive model was determined by time-dependent receiver operating characteristic (ROC) analysis.

### Acquisition of BBR-pharmacological targets

Websites, including the Traditional Chinese Medicine Systems Pharmacology Database and Analysis Platform (https://old.tcmsp-e.com/tcmsp.php), Swiss Target Prediction (http://swisstargetprediction.ch/), Search Tool for Interacting Chemicals (http://stitch.embl.de/), A Bioinformatics Analysis Tool for Molecular Mechanism of Traditional Chinese Medicine (http://bionet.ncpsb.org.cn/batman-tcm/index.php), Drugbank (https://go.drugbank.com/), and Super-pred (https://prediction.charite.de/) were used to screen and collect the pharmacological targets of BBR. Overlapping candidate genes were identified and validated using the UniProt database (https://www.uniprot.org/).

### Enrichment analyses and network visualization of overlapping candidate genes

The inflammation-related genes in PAAD and the pharmacological targets of BBR were compared to identify overlapping targets. STRING (https://string-db.org/) was used to generate the protein interaction networks and Cytoscape (version 3.7.2) was performed to visualize. The overlapping targets were annotated by the Gene Ontology (GO, http://geneontology.org) [13] and Kyoto Encyclopedia of Genes and Genomes (KEGG, https://www.genome.jp/kegg/) [14] databases, then plot the bar and bubble charts. Survival analysis of the four key targets was performed using the R packages (version 4.2.1). Finally, a drug–target pathway disease diagram was constructed using Cytoscape.

### Molecular docking simulation

The interaction between a small ligand molecule and an acceptor protein macromolecule can be simulated by molecular docking and their affinities can be predicted by calculate the binding energies between the two counterparts. PubChem (https://pubchem.ncbi.nlm.nih.gov/) provided the molecular structure of BBR, while the Protein Data Bank (PDB, https://www.rcsb.org/) provided the structures of the four key targets. AutoDockTools (version 1.5.6) was used to process the molecular docking, the specific docking process refer to the literature [15]. PyMol software (version 2.5.5) was used to visualize the molecular docking diagram.

### Cell culture and in vitro experiments

We obtained the pancreatic cancer cell line PANC-1 and Panc-02 cells from the American Type Culture Collection (Manassas, VA, USA) and the Frederick National Laboratory (Frederick, VA, USA) respectively. Cell culture was performed as described in reference 7. We purchased BBR chloride from Sigma-Aldrich (St. Louis, MO, USA). Cell viability, clonogenicity, western blotting, and cell cycle analyses were performed as described previously [7]. The following antibodies were used: anti-COX2, (diluted 1:1000, Cell Signaling Technology, Beverly, MA, USA), anti-CXCR4, anti-ICAM1 (diluted 1:1000, Proteintech, Wuhan, China), and anti-Caspase3 (diluted 1:1000, Abclonal, Wuhan, China). GAPDH (diluted1:10000, Proteintech) was used as an internal control.

### Animal model establishment and interventions

We obtained four- to six-week-old male C57BL/6 mice from Shanghai Jihui Laboratory Animal Care Co., Ltd. (Shanghai, China). Mice were housed in a specific pathogen-free environment with food and water provided *ad libitum*. We injected mouse Panc02 cells (2 × 10^6^) into C57BL/6 mice to establish the subcutaneous tumor model, as previously described [7]. Mice were randomized (*n* = 6) into three groups after tumor implantation and then saline or different doses of BBR were injected intraperitoneally into mice every 2 days for 28 days. Every 3 days we measured the mice weight and tumor size. Thirty-five days after implantation, the mice were euthanized through CO_2_ inhalation. Surgically dissected tumors were weighed and fixed in 4% paraformaldehyde. Animal experiments followed the guidelines for the care and use of laboratory animals and were approved by the Institutional Animal Care and Use Committee of Fudan University (Ethical Approval Number: 202308024Z).

### Hematoxylin and eosin staining and immunohistochemistry

Hematoxylin and eosin staining and immunohistochemistry (IHC) was performed as previously described [7]. Slides were observed under a microscope (Leica Microsystems Digital Imaging, Wetzlar, Germany). The antibodies used in this part were as follows: anti-COX2 (diluted 1:200, Cell Signaling Technology), anti-CXCR4, anti-ICAM1, anti-Ki-67 (diluted1:200, Proteintech), and anti-Caspase3 (diluted 1:200, Abclonal).

### Statistical analysis

All assays were performed independently in triplicate unless otherwise specified and the data were presented as the mean ± SD. The figure legends showed the statistical details of each experiment. One-way analysis of variance (ANOVA) and student’s t-test were used to analyze data using SPSS 21 (SPSS Inc., Chicago, IL, USA). *P*-value < 0.05 means that differences between means were considered significant.

## Results

### Clinical significance of inflammation-related genes in PAAD

**Fig. 2:**
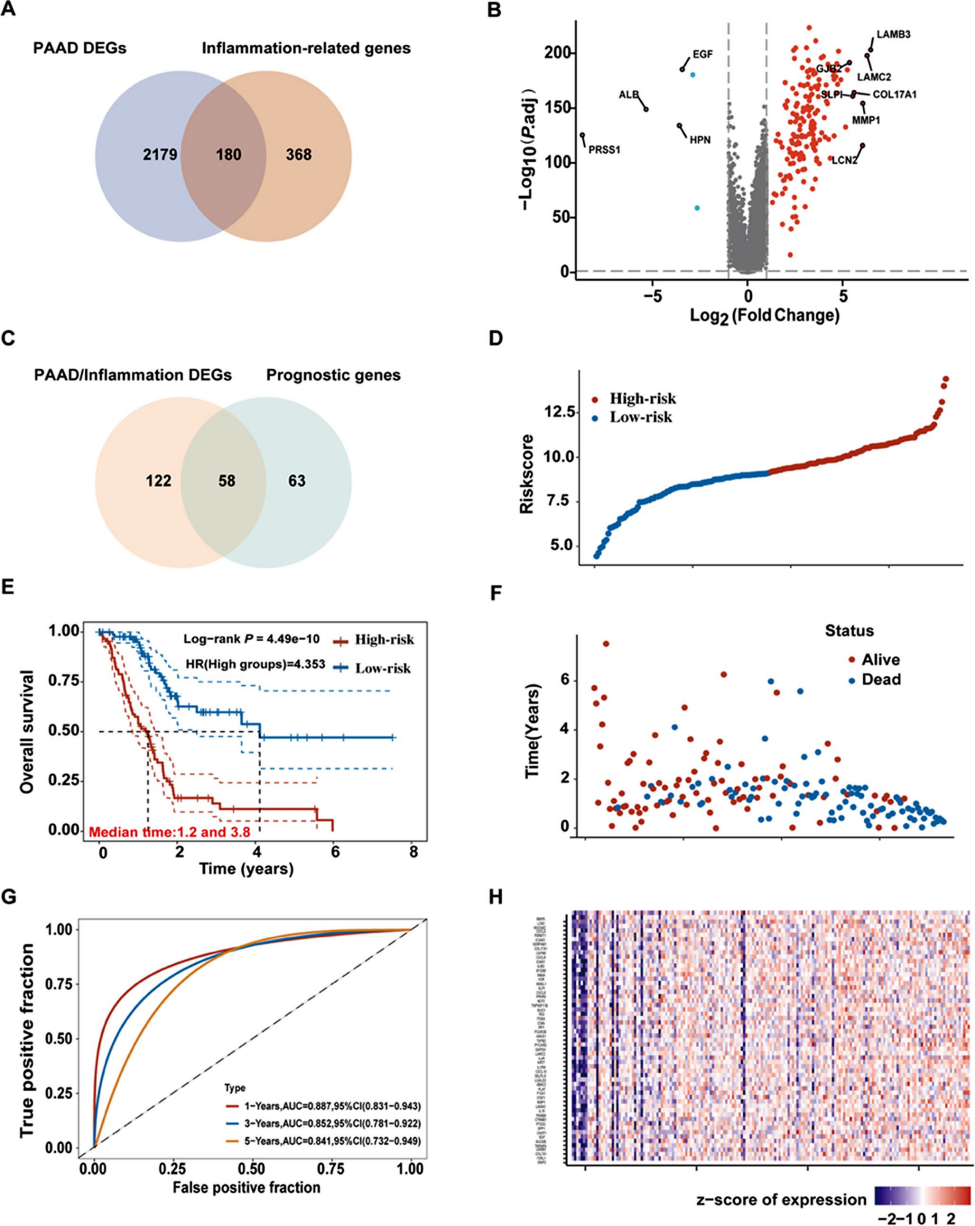
Prognostic significance of PAAD/inflammation. (**A**) Venn diagram to identify the inflammation- related genes in PAAD. (**B**) Volcano-plot representation of differential inflammation-related genes expression in PAAD. (**C**) Venn diagram to identify the prognostic genes in PAAD/inflammation. (**D**) Curve of risk score for the high- and low-risk groups. (**E**) Kaplan–Meier curves for OS of patients in high- and low-risk groups. (**F**) Scatter plot indicated the survival status of the high- and low-risk groups. (**G**) Time-dependent ROC analysis the prognostic-gene signature. (**H**) Heatmap showing expression profiles of the prognostic genes. *P* < 0.05 indicates significance. PAAD, pancreatic adenocarcinoma; BBR, berberine; DEGs, differentially expressed genes; AUC, the area under the curve; CI, confidence interval; ROC, receiver operating characteristic

First, 2359 DEGs in PAAD were obtained from TCGA and GTEx databases using GEPIA2. Additionally, 200 and 390 inflammation-related genes were obtained from the MSigDB and Genecards databases, respectively. All inflammation-related genes are listed in Supplementary Table 1. One hundred and eighty inflammation-related genes in PAAD were identified by comparing these two gene clusters (Fig. 2A). Then we observed that 174 genes were upregulated and six were downregulated in PAAD by examining the differential expression of the intersecting genes (Fig. 2B). Subsequently, 58 inflammation-related genes correlated with PAAD OS were identified as prognostic indicators by univariate Cox analysis (Fig. 2C, Supplementary Table 2). Multifactorial Cox regression analysis were used to construct prognostic models for identifying genes significantly associated with the prognosis of patients with PAAD. The risk scores are presented in Supplementary Table 3. According to the median cut-off value, patients were divided into high- and low-risk groups and survival analysis showed that high-risk patients had a significantly poorer survival than their low-risk counterparts (Fig. 2E); a gradual increase in deaths with increasing risk scores as showed in the risk curves and scatter plots (Fig. 2D and F). The sensitivity and specificity of the prediction model were analyzed by generating the time-dependent ROC curves; the area under the curve reached 0.887, 0.852, and 0.841 at 1, 3, and 5 years, respectively (Fig. 2G), indicating the high reliability of the model. Additionally, the prognostic genes expression profiles were displayed (Fig. 2H). These results suggested that inflammation-related genes are closely associated with PAAD prognosis and may provide new therapeutic targets for PAAD.

### Functional analysis of targets underlying BBR inhibition of PAAD/Inflammation

**Fig. 3:**
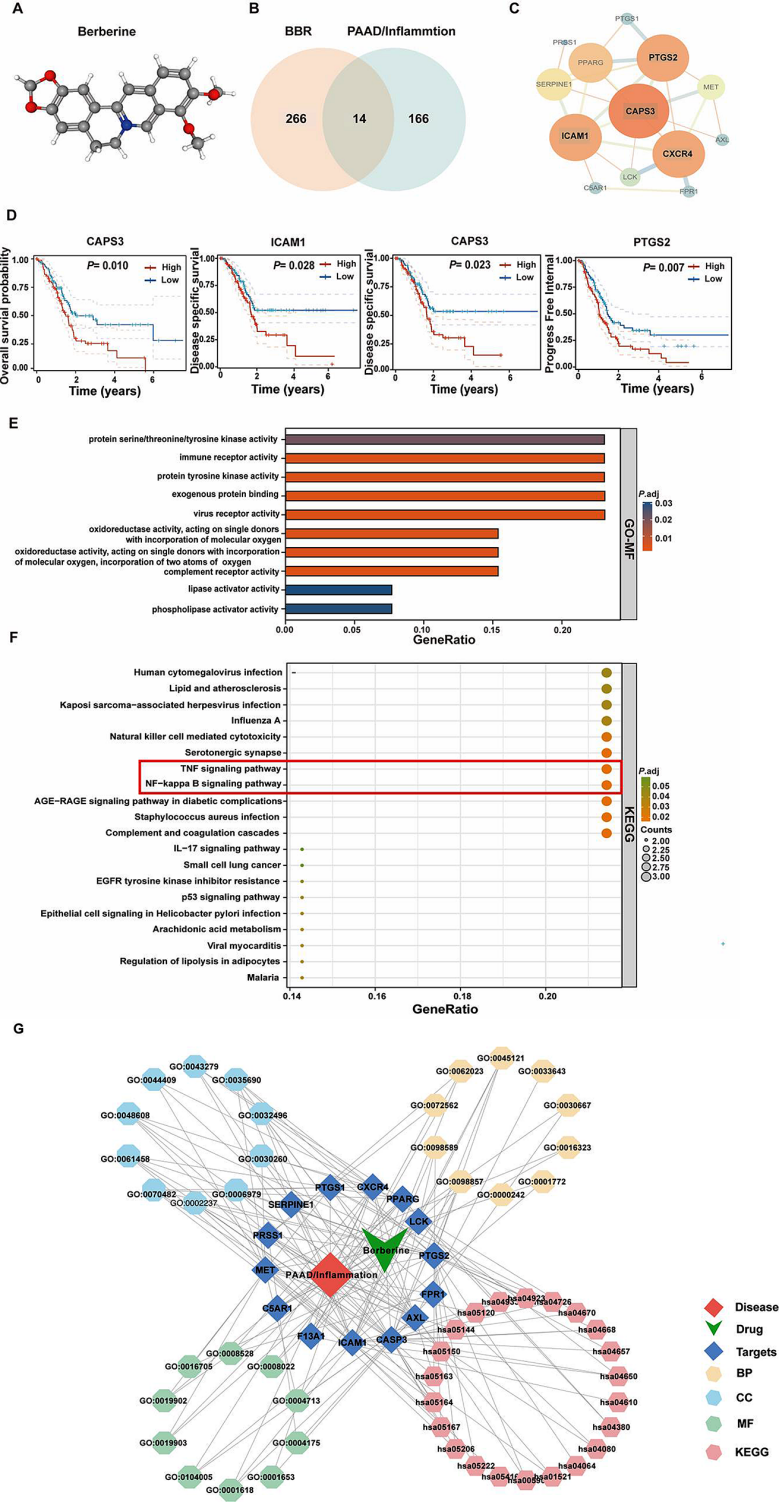
Functional analysis of targets that BBR inhibits in PAAD/inflammation. (**A**) 3D structure of Berberine. (**B**) Venn diagram of 14 overlapping targets for PAAD/inflammation and BBR. (**C**) PPI analysis screening for key targets-CAPS3, CXCR4, ICAM1, and PTGS2. (**D**) Survival analysis revealed differences in OS, PFI, and DSS according to the optimal cut-off expression value. (**E**) GO analysis of targets of BBR and PAAD/inflammation based on MF, which were mainly enriched in oxidoreductase activity and immune receptor activity. (**F**) KEGG analysis of top 20 pathways, including NF-kappa B signaling pathway and TNF signaling pathway. (**G**) Drug–target–pathway–disease network. *P* < 0.05 indicates significance; PAAD, pancreatic adenocarcinoma, BBR, berberine, GO-MF, gene ontology-molecular function; KEGG, Kyoto Encyclopedia of Genes and Genomes; OS, overall survival; PFI, progress free interval; DSS, disease specific survival; PPI, protein–protein interaction

The 3D structure of BBR is shown in Fig. 3A. Two-hundred-eighty BBR pharmacological targets were obtained from multiple databases, as described in the Methods section, and 14 intersecting genes were identified after an overlap of inflammation-related genes in PAAD with BBR-associated pharmacological targets (Fig. 3B). Protein–protein interaction (PPI) networks were illustrated using STRING, and then input the mapped intersecting proteins into the Cytoscape software to calculate the topological parameters, four key targets were highlighted: caspase 3 (CAPS3), Prostaglandin-Endoperoxide Synthase 2 (PTGS2), Intercellular Adhesion Molecule 1 (ICAM1), and C-X-C Motif Chemokine Receptor 4 (CXCR4) (Fig. 3C). Survival analysis revealed differences in OS, progression-free interval, and disease-specific survival between the high- and low-expression groups for the four key targets (Fig. 3D, *P* < 0.05).

Subsequently, the 14 intersecting genes were subjected to GO enrichment analyses, which indicated that BBR affects a series of molecular functions, cellular components, and biological processes, including response to lipopolysaccharide, response to molecules of bacterial origin, cellular response to drugs, response to oxygen levels, reproductive structure development, reproductive system development, response to oxidative stress, virus receptor activity, hijacked molecular function, protein tyrosine kinase activity, and protein phosphatase binding (Fig. 3E and S1). Additionally, 24 pathways related to all core targets were found in KEGG pathway analysis (*P*-adjust < 0.05), which included the nuclear factor kappa-B (NF-κB) signaling pathway, TNF signaling pathway, *staphylococcus aureus* infection, human cytomegalovirus infection, microRNAs in cancer, serotonergic synapse, natural killer cell mediated cytotoxicity, Influenza A, and kaposi sarcoma-associated herpesvirus infection (Fig. 3F). Figure 3G showed the drug–target–pathway–disease network.

### Molecular docking simulation

**Fig. 4:**
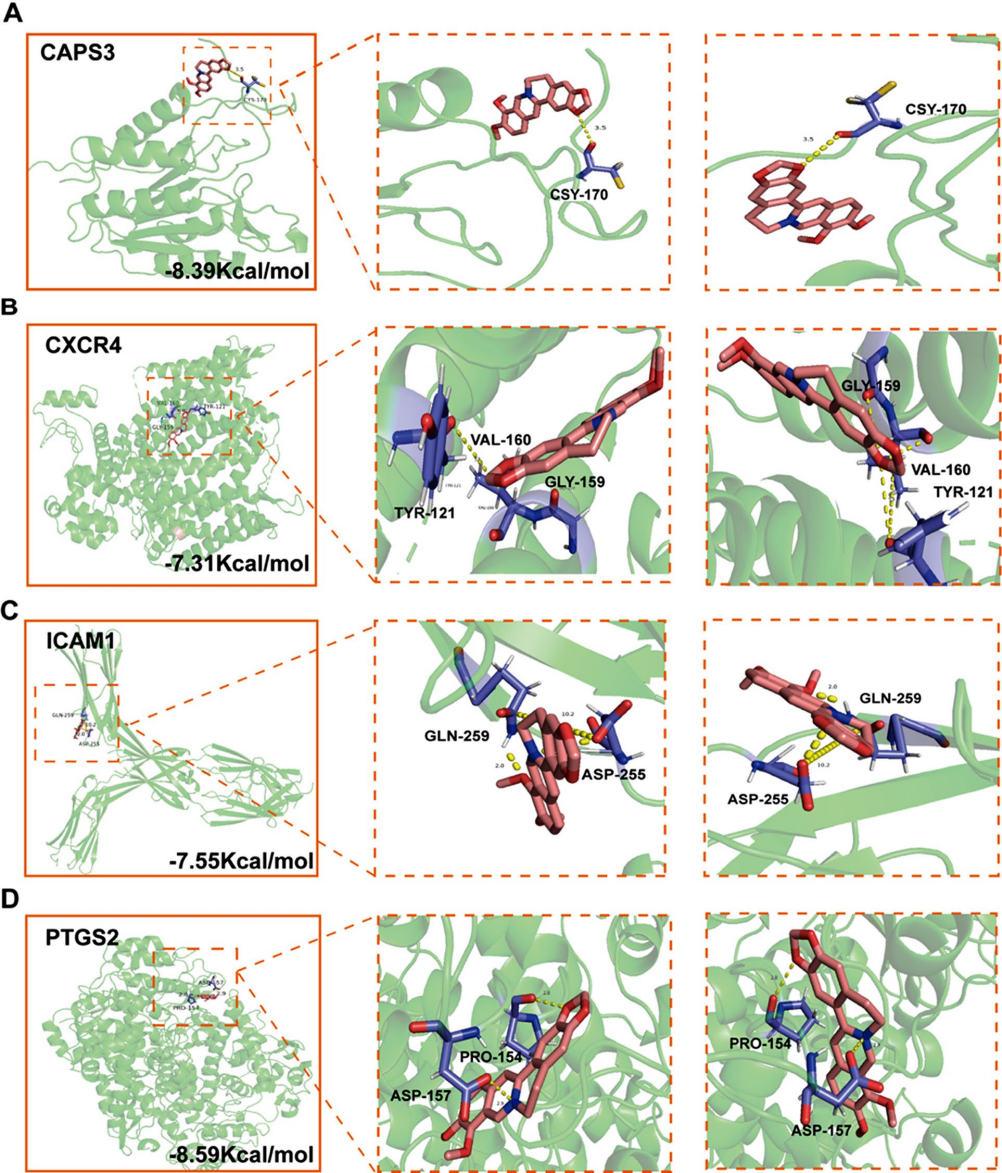
Molecular docking simulation and visualization of BBR binding with key targets, CAPS3, CXCR4, ICAM1, and PTGS2. (**A**) Sites of BBR binding with CAPS3 (binding energy − 8.39 Kcal/mol, hydrogen bond formed on CSY-170). (**B**) Sites of BBR binding with CXCR4 (binding energy − 7.31 Kcal/mol, hydrogen bond formed on TYR-121, VAL-120, and GLY-159). (**C**) Sites of BBR binding with ICAM1 (binding energy − 7.55 Kcal/mol, hydrogen bond formed on ASP-255 and GLN-259). (**D**) Sites of BBR binding with PTGS2 (binding energy − 8.59 Kcal/mol, hydrogen bond formed on PRO-154 and ASP-157). BBR, berberine

Molecular docking analysis was performed to determine the possible binding of BBR to the identified key targets. The crystal structures of the four targets, CAPS3 (PDB ID 1NME), PTGS2 (PDB ID 5IKV), ICAM1 (PDB ID 1P53), and CXCR4 (PDB ID 3OE9), were retrieved from the PDB database for docking stimulation against BBR. Binding energies below zero indicated that the two molecules bound spontaneously, with smaller binding energies leading to a more stable conformation [16]. The results showed that the four key targets docked well with BBR; the structures and binding sites are shown in Fig. 4.

### BBR suppressed PAAD cell proliferation in vitro

**Fig. 5:**
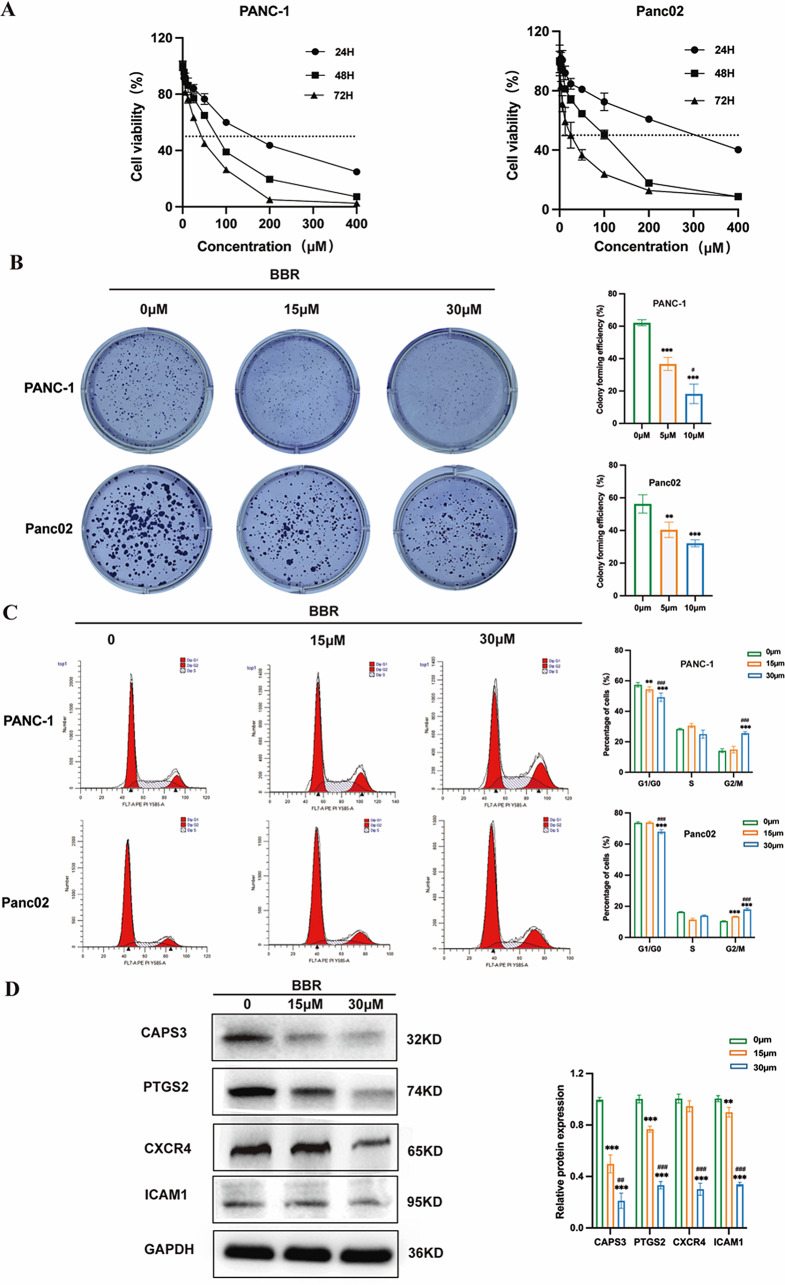
BBR suppressed PAAD proliferation in vitro. (**A**) Cell viability assays after intervention of BBR. (**B**) Cell clonogenicity assays after intervention of BBR. (**C**) Cell cycle analysis after intervention of BBR. (**D**) The key protein expression after intervention of BBR. BBR, berberine; PAAD, pancreatic adenocarcinoma. *P* < 0.05 indicates significance: *P < 0.05, ** *P <* 0.01, ***P < 0.001 when compared with the control group; # *P* < 0.05, ## *P* < 0.01 and ### *P* < 0.001 when compared with the 5 µM group

We further verified the inhibitory effects of BBR on PAAD cells in vitro. The cell viability, determined using the CCK8 assay, showed that after intervention for 24, 48, and 72 h, the PANC-1 cells respective IC50 values were 146.1 µM, 68.89 µM, and 36.63 µM, and the Panc02 cells respective IC50 values were 284.7 µM, 73.75 µM, and 22.33 µM, means that BBR inhibited PAAD cells dose-dependently and time-dependently (Fig. 5A). Results of the colony formation assay suggested that as the drug concentration increased, the colony formation ability was considerably inhibited (Fig. 5B). Subsequently, we investigated whether BBR had an effect on cell cycle arrest in PAAD cells; after incubation with 0, 15, and 30 µM BBR for 48 h, the results showed that the percentage of G2/M phase cells increased from 14.19 ± 1.24% to 25.71 ± 0.93% in PANC-1 and from 10.59 ± 0.069% to 18.09 ± 0.39% in Panc02 cells (Fig. 5C). These results suggest that in vitro BBR inhibited PAAD cell proliferation and induced cell cycle arrest.

### BBR suppressed PAAD growth in vivo

**Fig. 6:**
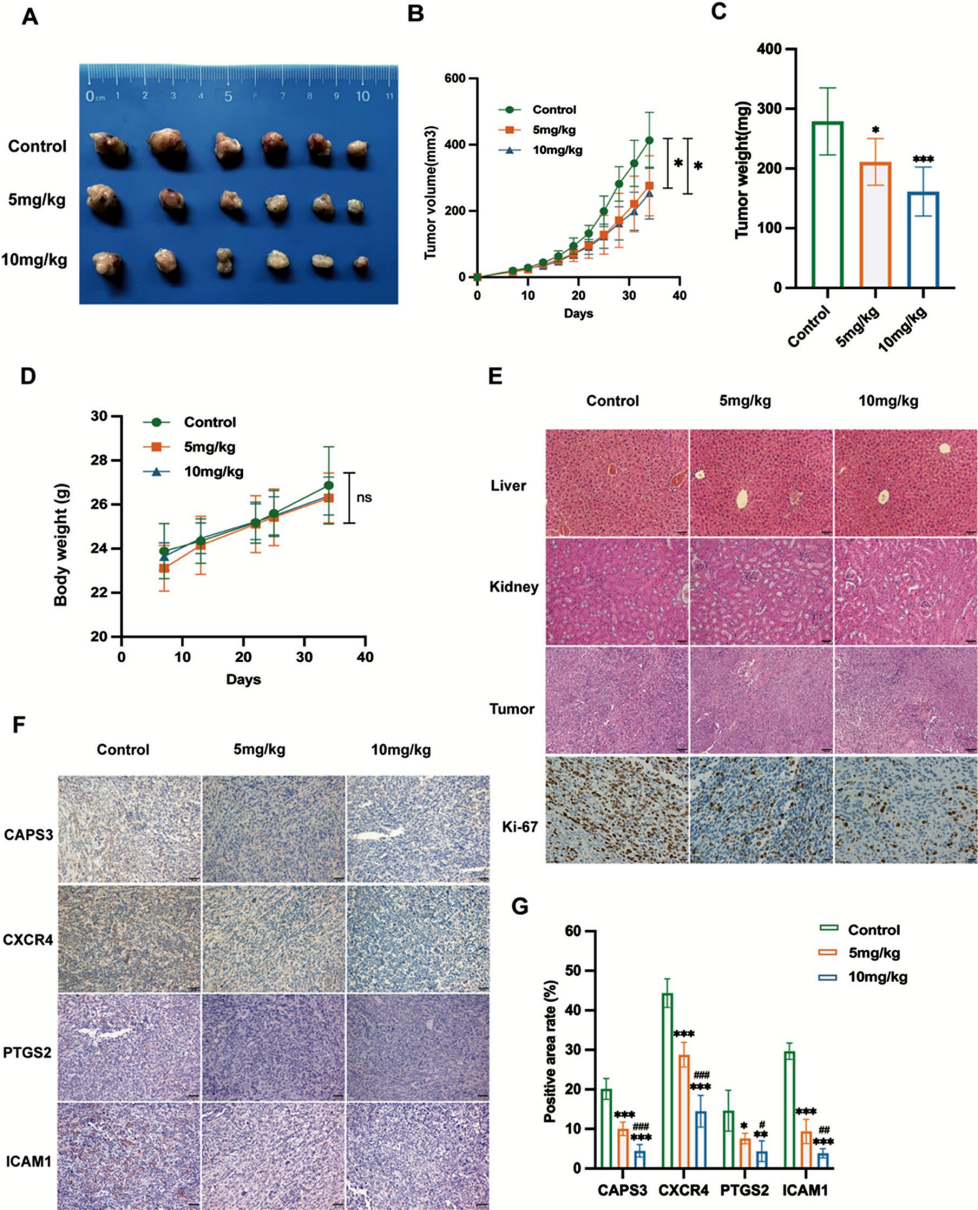
BBR inhibits pancreatic tumor growth in vivo. (**A**) Tumors in different groups of mice (*n* = 6). (**B**) Tumor growth curve. (**C**) Tumor weights between different groups. (**D**) Changes in mouse body weight. (**E**) Representative images of HE staining in the liver, kidney, and tumor tissues (HE, 100×, 200×), and Ki-67 (IHC, 200×). (**F**). Immunohistochemistry analysis and statistical analysis of the effects of BBR on CAPS3, PTGS2, CXCR4, and ICAM1 expression (IHC, 200×). BBR, berberine; HE, hematoxylin and eosin; IHC, immunohistochemistry; *P* < 0.05 indicates significance: *P < 0.05, ** *P <* 0.01, ***P < 0.001 when compared with the control group; # *P* < 0.05, ## *P* < 0.01 and ### *P* < 0.001 when compared with the 5 mg/kg group

A pancreatic subcutaneous tumor model was established to investigate whether BBR inhibits PAAD in vivo. Different doses of BBR were injected intraperitoneally for 28 days. The results show that compared with the control group, BBR significantly reduced tumor volume, tumor growth rate, and tumor weight (Fig. 6A–C, *P* < 0.05). Higher proportion of Ki-67-positive cells were observed in the control group than in the BBR treat group, indicating that BBR suppressed the proliferative activity of the tumor (Fig. 6E). Furthermore, there was no significant decrease in mice body weight (Fig. 6D, *P* > 0.05) or pathological changes upon histological examination of the liver or kidneys (Fig. 6E) after BBR intervention. Taken together, our results suggest that BBR suppresses tumor growth in mouse PAAD models.

### BBR suppressed inflammation-related gene protein expression

Next, we verified whether BBR inhibited PAAD via the four key targets. IHC results demonstrated that, treatment with 5 and 10 mg/kg of BBR significantly suppressed the protein expression of the four candidate targets in vivo compared with the control group (Fig. 6F, *P* < 0.01). Meanwhile, western blotting results showed that the four candidate targets protein expression were reduced after treatment with different does of BBR, among which CAPS3 and PTGS2 were significant in vitro (Fig. 5D). These results demonstrated that BBR inhibits PAAD may be associated with suppress the inflammation-related genes.

## Discussion

PAAD is a deadly malignancy, and > 50% of patients have metastasis at the time of diagnosis [1]. Very few patients benefit from surgery, and the first-line chemotherapy regimens using 5-fluorouracil, calcium folinate, irinotecan, and oxaliplatin (FOLFIRONOX) and gemcitabine plus albumin-bound paclitaxel do not produce a significant survival benefit [17]. The rising incidence and persistently poor 5-year OS of patients with PAAD highlight the need for new effective therapies.

Inflammation is emerging as a hallmark of cancer that promotes cancer development and progression [18]. Overexpression of inflammatory factors promotes tumor development, while targeting inflammatory mediators, such as chemokines, cytokines (TNF-α and IL-1β), and key transcription factors (NF-κB and STAT3), reduces cancer growth and spread [19]. Therefore, inflammation is a promising therapeutic target in PAAD. In contrast to previous studies, we focused on inflammation-related genes to identify novel mechanisms and targets. In the present study, we systematically analyzed the expression of 548 inflammation-related genes in PAAD tissues and their association with OS. One hundred and eighty DEGs were screened; 58 DEGs were revealed associated with OS by univariate Cox analysis. Subsequently, an inflammation-related prognostic risk model with strong predictive power was established using a multifactor Cox regression analysis. Patients were categorized into high- and low-risk groups according to the median risk score, and the high-risk group was significantly associated with worse OS. Another study including 200 inflammation-related genes confirmed that these genes are not only used to predict prognosis but also affect the immune status of PAAD [20]. Our results indicate that inflammation-related genes in PAAD are closely correlated with prognosis, and suppression of these genes may be a promising treatment option.

BBR is widely used for the treatment of various diseases, including inflammation and tumors. However, the specific mechanisms underlying its targeting of PAAD warrant further clarification. In the present study, we aimed to clarify whether BBR suppresses PAAD by targeting inflammation-related genes. By intersecting 180 genes related to inflammation in PAAD with 260 pharmacological targets of BBR, we identified 14 overlapping pharmacological targets. PPI analysis showed that CAPS3, PTGS2, ICAM1, and CXCR4 were the key genes affected by BBR in PAAD. CAPS3 plays a vital role as an effector in the apoptosis pathway, initiating through precursor-mediated cleavage that methodically dismantles cellular structure, ultimately leading to cell death [21]. ICAM-1, a cell surface glycoprotein, regulates the behavior of tumor cells by facilitating interactions with the cellular microenvironment and the extracellular matrix [22]. CXCR4, a member of the G protein-coupled receptor (GPCR) family, controls a range of cellular functions, such as tumor cell migration, proliferation, and survival [23]. The enzyme PTGS2, which plays a crucial role in inflammation and tumorigenesis, is implicated in promoting the progression of malignancy by enhancing tumor invasiveness and evading apoptotic pathways [24]. The proteins CAPS3, ICAM-1, CXCR4, and PTGS2 are key players in regulating the life cycles of tumor cells. BBR downregulates these proteins, indicating a potential mechanism through which BBR inhibits tumor growth by modulating inflammation-related genes. Enrichment analysis reveals that BBR targets are primarily associated with the TNF and NF-κB signaling pathways, which play crucial roles in regulating inflammation, immune responses, and oncogenic progression [25, 26]. Existing research provides evidence of BBR’s anti-inflammatory and potential anti-tumor effects through these pathways [27, 28]. These molecular interactions collectively support the proposed mechanism of action of BBR in the treatment of PAAD.

Next, we validated the results in vitro and in vivo. Cell proliferation and cell cycle analyses performed in vitro showed that BBR effectively inhibited the proliferation of PAAD cells. Subsequently, we investigated the effect of BBR on PAAD in vivo and found that BBR exhibited significant tumor suppression in a mouse subcutaneous tumor model without reducing body weight, and no significant damage was observed in the pathological sections of the liver or kidney. Previous studies have documented the suboptimal gastrointestinal absorption and rapid systemic metabolism of BBR, factors that contribute to its reduced bioavailability [29]. Consequently, we used an intraperitoneal injection method for this in vivo study. Dose levels of 5 mg/kg and 10 mg/kg were established for our experimental design, drawing upon extant research findings and clinical dosages of BBR in human subjects [7]. Furthermore, there was no significant decrease in mice body weight or pathological changes upon histological examination of the liver or kidneys after BBR intervention, implied that the mice demonstrated a favorable tolerance to the selected method and dosage of BBR administration. Despite the 10 mg/kg dosage exhibiting a trend towards tumor volume and tumor weight reduction in comparison to the 5 mg/kg dosage, the disparity did not attain statistical significance. We surmise that the dosages, while within a safe threshold, have not reached an efficacy plateau. To elucidate the dose-response relationship with greater clarity, future research will broaden the dosing spectrum to ascertain BBR’s minimum effective dose and its ceiling of safety.

IHC and western blotting showed that BBR decreased the protein expression of the four key targets in vitro and vivo, suggesting that BBR suppresses PAAD by targeting inflammation-related genes. Moreover, our findings suggest a decrease in the expression levels of the CAPS3 precursor protein, a key component in the apoptotic pathway. This observation contradicts the pro-apoptotic effects of BBR on tumor cells. The proteolytic cleavage of the CAPS3 precursor during apoptosis leads to its activation, which plays a crucial role in promoting cell death. Therefore, the decrease in CAPS3 precursor expression may indicate activation of CAPS3, suggesting that BBR may induce apoptosis by promoting CAPS3 activation. Furthermore, the orchestration of apoptosis involves a complex regulatory network consisting of diverse signaling pathways and molecular components. The mechanistic effects of BBR on apoptosis extend beyond the activation of CAPS3, demonstrating the intricate and multifaceted nature of apoptotic regulation. This emphasizes the importance of gaining a comprehensive understanding of the various roles of BBR in modulating cell death mechanisms. Our study exclusively validated key targets without exploring inflammation-related pathways, and future studies should consider incorporating inflammation inhibitors and agonists.

## Conclusion

This study successfully identified the prognostic significance of inflammation-related genes in PAAD and validated the inhibitory effects of BBR on PAAD by targeting these genes. However, it is important to note that the genes examined in this study were obtained from databases, and future investigations should include the validation of gene expression alterations before and after treatment using actual clinical samples. The present study only verified the expression of key inflammation-related genes after BBR intervention, and whether BBR affect inflammation-related pathways and phenotypes needs to be investigated in depth in subsequent studies. Additionally, large-scale preclinical and clinical trials are needed to establish stronger evidence to support the clinical use of BBR for PAAD.

## Electronic supplementary material

Below is the link to the electronic supplementary material.

Supplementary Material 1

## Data Availability

The authors confirm that the data supporting the findings of this study are available within the article and its supplementary materials.
