# Supplementary material for: Berberine chloride suppresses pancreatic adenocarcinoma proliferation and growth by targeting inflammation-related genes: an in silico analysis with in vitro and vivo validation
Source: Cancer Chemother Pharmacol. 2024 Mar 19;94(2):169–81. doi: 10.1007/s00280-024-04663-7 (PMC11390897; doi:10.1007/s00280-024-04663-7)
Supplement: Supplementary file 1 — Supplementary Material 1 [file 280_2024_4663_MOESM1_ESM.docx]

1. Supplementary Table 1: Inflammation-related genes

| \| Genecard database \| the Molecular Signatures database \| \| --- \| --- \| \| NLRP3 \| ABCA1 \| \| IL6 \| ABI1 \| \| IL10 \| ACVR1B \| \| TNF \| ACVR2A \| \| CRP \| ADM \| \| TLR4 \| ADORA2B \| \| SYK \| ADRM1 \| \| NOD2 \| AHR \| \| IL1B \| APLNR \| \| CXCL8 \| AQP9 \| \| MEFV \| ATP2A2 \| \| HLA-DRB1 \| ATP2B1 \| \| HLA-B \| ATP2C1 \| \| TGFB1 \| AXL \| \| CTLA4 \| BDKRB1 \| \| PTGS2 \| BEST1 \| \| TNFRSF1A \| BST2 \| \| IL17A \| BTG2 \| \| IL13 \| C3AR1 \| \| CCL2 \| C5AR1 \| \| TLR2 \| CALCRL \| \| PRTN3 \| CCL17 \| \| IL1RN \| CCL2 \| \| IFNG \| CCL20 \| \| STAT1 \| CCL22 \| \| PLG \| CCL24 \| \| FOXP3 \| CCL5 \| \| ELANE \| CCL7 \| \| NFKB1 \| CCR7 \| \| IL4 \| CCRL2 \| \| PTPN22 \| CD14 \| \| ICAM1 \| CD40 \| \| IL18 \| CD48 \| \| FAS \| CD55 \| \| STAT3 \| CD69 \| \| TNFAIP3 \| CD70 \| \| ALB \| CD82 \| \| HMGB1 \| CDKN1A \| \| IL1A \| CHST2 \| \| MMP9 \| CLEC5A \| \| MPO \| CMKLR1 \| \| MMP1 \| CSF1 \| \| IRF5 \| CSF3 \| \| ILRUN \| CSF3R \| \| RIPK1 \| CX3CL1 \| \| CCL11 \| CXCL10 \| \| RELA \| CXCL11 \| \| IL23R \| CXCL6 \| \| MIF \| CXCL9 \| \| STAT4 \| CXCR6 \| \| ITGAM \| CYBB \| \| IL10RA \| DCBLD2 \| \| VCAM1 \| EBI3 \| \| VEGFA \| EDN1 \| \| IL2 \| EIF2AK2 \| \| SELE \| EMP3 \| \| IL37 \| ADGRE1 \| \| IL12A \| EREG \| \| RNASE3 \| F3 \| \| LTA \| FFAR2 \| \| JAK1 \| FPR1 \| \| PPARG \| FZD5 \| \| HLA-DQB1 \| GABBR1 \| \| CCR6 \| GCH1 \| \| INS \| GNA15 \| \| CCR1 \| GNAI3 \| \| ITGB2 \| GP1BA \| \| IL5 \| GPC3 \| \| CCL5 \| GPR132 \| \| CSF2 \| GPR183 \| \| SAA1 \| HAS2 \| \| HLA-DPB1 \| HBEGF \| \| CCR5 \| HIF1A \| \| ABCB1 \| HPN \| \| PSTPIP1 \| HRH1 \| \| ADIPOQ \| ICAM1 \| \| ALOX5 \| ICAM4 \| \| SPP1 \| ICOSLG \| \| CASP1 \| IFITM1 \| \| CFTR \| IFNAR1 \| \| CXCL10 \| IFNGR2 \| \| SERPINA1 \| IL10 \| \| HLA-DQA1 \| IL10RA \| \| IRAK1 \| IL12B \| \| APOE \| IL15 \| \| FLG \| IL15RA \| \| C4A \| IL18 \| \| OTULIN \| IL18R1 \| \| ADA2 \| IL18RAP \| \| S100A9 \| IL1A \| \| CARD8 \| IL1B \| \| NOS2 \| IL1R1 \| \| FASLG \| IL2RB \| \| AGER \| IL4R \| \| IFNGR1 \| IL6 \| \| IL12RB1 \| IL7R \| \| CD4 \| CXCL8 \| \| ABCB4 \| INHBA \| \| IL1R1 \| IRAK2 \| \| IKBKG \| IRF1 \| \| STING1 \| IRF7 \| \| HMOX1 \| ITGA5 \| \| IL2RA \| ITGB3 \| \| LEP \| ITGB8 \| \| RETN \| KCNA3 \| \| NFKBIA \| KCNJ2 \| \| DEFB4A \| KCNMB2 \| \| SELP \| KIF1B \| \| CHI3L1 \| KLF6 \| \| PDCD1 \| LAMP3 \| \| NLRP1 \| LCK \| \| IRGM \| LCP2 \| \| CIITA \| LDLR \| \| TLR3 \| LIF \| \| BTNL2 \| LPAR1 \| \| F2 \| LTA \| \| TP53 \| LY6E \| \| CD40LG \| LYN \| \| FCGR2A \| MARCO \| \| CCL3 \| MEFV \| \| HFE \| MEP1A \| \| ERAP1 \| MET \| \| S100A8 \| MMP14 \| \| KNG1 \| MSR1 \| \| PRKCQ \| MXD1 \| \| PTX3 \| MYC \| \| MMP3 \| NAMPT \| \| EGFR \| NDP \| \| C3 \| NFKB1 \| \| HLA-DPA1 \| NFKBIA \| \| HLA-A \| NLRP3 \| \| IKBKB \| NMI \| \| MAPK14 \| NMUR1 \| \| IL10RB \| NOD2 \| \| DNASE1 \| NPFFR2 \| \| MYD88 \| OLR1 \| \| TREX1 \| OPRK1 \| \| NLRP12 \| OSM \| \| CXCL1 \| OSMR \| \| AKT1 \| P2RX4 \| \| CP \| P2RX7 \| \| CD40 \| P2RY2 \| \| CXCL2 \| PCDH7 \| \| PTGS1 \| PDE4B \| \| CXCR3 \| PDPN \| \| PLA2G7 \| PIK3R5 \| \| MVK \| PLAUR \| \| IL12B \| PROK2 \| \| TSLP \| PSEN1 \| \| ACE \| PTAFR \| \| SERPINE1 \| PTGER2 \| \| SERPINA3 \| PTGER4 \| \| CYBB \| PTGIR \| \| LCN2 \| PTPRE \| \| HP \| PVR \| \| TNFSF11 \| RAF1 \| \| TNIP1 \| RASGRP1 \| \| NFE2L2 \| RELA \| \| LACC1 \| RGS1 \| \| IL33 \| RGS16 \| \| CYBC1 \| RHOG \| \| TNFSF15 \| RIPK2 \| \| TBX21 \| RNF144B \| \| PIK3CD \| ROS1 \| \| UBA1 \| RTP4 \| \| CYBA \| SCARF1 \| \| SELL \| SCN1B \| \| NLRC4 \| SELE \| \| C4B \| SELL \| \| IFIH1 \| SELENOS \| \| XIAP \| SEMA4D \| \| NOD1 \| SERPINE1 \| \| MMP2 \| SGMS2 \| \| NFKB2 \| SLAMF1 \| \| IL2RB \| SLC11A2 \| \| IL6ST \| SLC1A2 \| \| DNASE1L3 \| SLC28A2 \| \| NCF4 \| SLC31A1 \| \| COL7A1 \| SLC31A2 \| \| PPARA \| SLC4A4 \| \| FCGR3B \| SLC7A1 \| \| CSF3 \| SLC7A2 \| \| MST1 \| SPHK1 \| \| IL21 \| SRI \| \| CD14 \| STAB1 \| \| TLR9 \| TACR1 \| \| MUC5B \| TACR3 \| \| ADRB2 \| TAPBP \| \| PIK3CG \| TIMP1 \| \| IL4R \| TLR1 \| \| TTR \| TLR2 \| \| IL36RN \| TLR3 \| \| THBD \| TNFAIP6 \| \| MAPK1 \| TNFRSF1B \| \| TNFRSF1B \| TNFRSF9 \| \| PIK3CA \| TNFSF10 \| \| MUC1 \| TNFSF15 \| \| TRAF6 \| TNFSF9 \| \| NCF1 \| TPBG \| \| CR2 \| VIP \| \| FERMT1 \|  \| \| FCGR2B \|  \| \| SPINK1 \|  \| \| CXCL12 \|  \| \| ETS1 \|  \| \| FGFR2 \|  \| \| CD28 \|  \| \| NCF2 \|  \| \| IL1RAPL2 \| \| \| ANXA1 \|  \| \| IL17F \|  \| \| IL6R \|  \| \| S100A12 \|  \| \| CD79A \|  \| \| CTSG \|  \| \| F2RL1 \|  \| \| LPL \|  \| \| SLC22A4 \| \| \| IL15 \|  \| \| CCR3 \|  \| \| VWF \|  \| \| TYK2 \|  \| \| PADI4 \|  \| \| CCL4 \|  \| \| MBTPS2 \|  \| \| CD80 \|  \| \| ENG \|  \| \| PLA2G2A \| \| \| TIMP1 \|  \| \| CASR \|  \| \| CXCL9 \|  \| \| NR1H4 \|  \| \| SEMA4D \| \| \| TLR1 \|  \| \| IGHE \|  \| \| WAS \|  \| \| IRF1 \|  \| \| TNNI3 \|  \| \| NR3C1 \|  \| \| PLCG2 \|  \| \| VDR \|  \| \| INAVA \|  \| \| F5 \|  \| \| IL9 \|  \| \| CASP3 \|  \| \| ATG16L1 \| \| \| TAB2 \|  \| \| CTNNB1 \|  \| \| PSMB8 \|  \| \| FGF10 \|  \| \| KLRC4 \|  \| \| MUC5AC \| \| \| CASP8 \|  \| \| PRKCD \|  \| \| CCL20 \|  \| \| LTF \|  \| \| SLPI \|  \| \| CX3CR1 \|  \| \| NR1H3 \|  \| \| ICOSLG \|  \| \| CD8A \|  \| \| UBAC2 \|  \| \| TCF4 \|  \| \| CALCA \|  \| \| GPT \|  \| \| PRF1 \|  \| \| EDN1 \|  \| \| GZMB \|  \| \| PRSS1 \|  \| \| JAK2 \|  \| \| LAMC2 \|  \| \| UBE2L3 \|  \| \| AIRE \|  \| \| BCL2 \|  \| \| NAMPT \|  \| \| MMP8 \|  \| \| SCGB1A1 \| \| \| SOCS1 \|  \| \| TNFRSF11B \| \| \| TLR8 \|  \| \| HAMP \|  \| \| GPR35 \|  \| \| MBL2 \|  \| \| SRC \|  \| \| CXCR2 \|  \| \| TNFSF4 \|  \| \| CCR7 \|  \| \| CD36 \|  \| \| ABCB11 \|  \| \| FGFR3 \|  \| \| SAMHD1 \| \| \| BPI \|  \| \| EPX \|  \| \| SERPINB1 \| \| \| FADD \|  \| \| PTGDR2 \|  \| \| TAC1 \|  \| \| HLA-G \|  \| \| PYCARD \| \| \| C5 \|  \| \| SOCS3 \|  \| \| CXCR4 \|  \| \| GJB2 \|  \| \| APOA1 \|  \| \| MMEL1 \|  \| \| GNE \|  \| \| SREBF1 \|  \| \| CASP10 \|  \| \| IL17RA \|  \| \| CD274 \|  \| \| ACKR2 \|  \| \| REL \|  \| \| JUN \|  \| \| TF \|  \| \| NR1H2 \|  \| \| CD86 \|  \| \| ADAR \|  \| \| SLC9A3 \|  \| \| RASGRP1 \| \| \| ZEB1 \|  \| \| HSPD1 \|  \| \| TNFSF12 \| \| \| TOM1 \|  \| \| SH2D1A \|  \| \| ADAM17 \| \| \| IFNA1 \|  \| \| CAPN5 \|  \| \| FPR2 \|  \| \| IL22 \|  \| \| TNFSF13B \| \| \| EGF \|  \| \| CD44 \|  \| \| LBP \|  \| \| CX3CL1 \|  \| \| SFTPD \|  \| \| HIF1A \|  \| \| SIRT1 \|  \| \| BDNF \|  \| \| HAVCR2 \| \| \| CSTB \|  \| \| MECP2 \|  \| \| TBK1 \|  \| \| ADA \|  \| \| TGFB2 \|  \| \| LTB4R \|  \| \| CRYAA \|  \| \| MMP13 \|  \| \| LAMA3 \|  \| \| SERPINC1 \| \| \| RARRES2 \| \| \| CHUK \|  \| \| CAT \|  \| \| ITGB4 \|  \| \| CD163 \|  \| \| SFTPC \|  \| \| TNFAIP6 \| \| \| CD55 \|  \| \| NOS3 \|  \| \| F3 \|  \| \| ALOX15 \|  \| \| F13A1 \|  \| \| PSMB4 \|  \| \| MTHFR \|  \| \| CSF1 \|  \| \| BACH2 \|  \| \| PON1 \|  \| \| HSD3B7 \|  \| \| IL7 \|  \| \| SAA4 \|  \| \| COL17A1 \| \| \| ARPC1B \|  \| \| MAPK8 \|  \| \| SLC37A4 \| \| \| MME \|  \| \| NPM1 \|  \| \| IGF1 \|  \| \| IL23A \|  \| \| TLR7 \|  \| \| LAMB3 \|  \| \| IL16 \|  \| \| GAPDH \|  \| \| CD19 \|  \| \| POLA1 \|  \| \| LPIN2 \|  \| \| SELPLG \|  \| \| PLAT \|  \| \| MUC7 \|  \| \| CCL13 \|  \| \| PI4KA \|  \| \| LGALS3 \|  \| \| CXCL5 \|  \| \| NPPB \|  \| \| POU2AF1 \| \| \| KRT7 \|  \| \| LRP1 \|  \| \| IKZF1 \|  \| \| AZU1 \|  \| \| IRF3 \|  \| \| BLK \|  \| |
| --- | --- | --- | --- | --- | --- | --- | --- | --- | --- | --- | --- | --- | --- | --- | --- | --- | --- | --- | --- | --- | --- | --- | --- | --- | --- | --- | --- | --- | --- | --- | --- | --- | --- | --- | --- | --- | --- | --- | --- | --- | --- | --- | --- | --- | --- | --- | --- | --- | --- | --- | --- | --- | --- | --- | --- | --- | --- | --- | --- | --- | --- | --- | --- | --- | --- | --- | --- | --- | --- | --- | --- | --- | --- | --- | --- | --- | --- | --- | --- | --- | --- | --- | --- | --- | --- | --- | --- | --- | --- | --- | --- | --- | --- | --- | --- | --- | --- | --- | --- | --- | --- | --- | --- | --- | --- | --- | --- | --- | --- | --- | --- | --- | --- | --- | --- | --- | --- | --- | --- | --- | --- | --- | --- | --- | --- | --- | --- | --- | --- | --- | --- | --- | --- | --- | --- | --- | --- | --- | --- | --- | --- | --- | --- | --- | --- | --- | --- | --- | --- | --- | --- | --- | --- | --- | --- | --- | --- | --- | --- | --- | --- | --- | --- | --- | --- | --- | --- | --- | --- | --- | --- | --- | --- | --- | --- | --- | --- | --- | --- | --- | --- | --- | --- | --- | --- | --- | --- | --- | --- | --- | --- | --- | --- | --- | --- | --- | --- | --- | --- | --- | --- | --- | --- | --- | --- | --- | --- | --- | --- | --- | --- | --- | --- | --- | --- | --- | --- | --- | --- | --- | --- | --- | --- | --- | --- | --- | --- | --- | --- | --- | --- | --- | --- | --- | --- | --- | --- | --- | --- | --- | --- | --- | --- | --- | --- | --- | --- | --- | --- | --- | --- | --- | --- | --- | --- | --- | --- | --- | --- | --- | --- | --- | --- | --- | --- | --- | --- | --- | --- | --- | --- | --- | --- | --- | --- | --- | --- | --- | --- | --- | --- | --- | --- | --- | --- | --- | --- | --- | --- | --- | --- | --- | --- | --- | --- | --- | --- | --- | --- | --- | --- | --- | --- | --- | --- | --- | --- | --- | --- | --- | --- | --- | --- | --- | --- | --- | --- | --- | --- | --- | --- | --- | --- | --- | --- | --- | --- | --- | --- | --- | --- | --- | --- | --- | --- | --- | --- | --- | --- | --- | --- | --- | --- | --- | --- | --- | --- | --- | --- | --- | --- | --- | --- | --- | --- | --- | --- | --- | --- | --- | --- | --- | --- | --- | --- | --- | --- | --- | --- | --- | --- | --- | --- | --- | --- | --- | --- | --- | --- | --- | --- | --- | --- | --- | --- | --- | --- | --- | --- | --- | --- | --- | --- | --- | --- | --- | --- | --- | --- | --- | --- | --- | --- | --- | --- | --- | --- | --- | --- | --- | --- | --- | --- | --- | --- | --- | --- | --- | --- | --- | --- | --- | --- | --- | --- | --- | --- | --- | --- | --- | --- | --- | --- | --- | --- | --- | --- | --- | --- | --- | --- | --- | --- | --- | --- | --- | --- | --- | --- | --- | --- | --- | --- | --- | --- | --- | --- | --- | --- | --- | --- | --- | --- | --- | --- | --- | --- | --- | --- | --- | --- | --- | --- | --- | --- | --- | --- | --- | --- | --- | --- | --- | --- | --- | --- | --- | --- | --- | --- | --- | --- | --- | --- | --- | --- | --- | --- | --- | --- | --- | --- | --- | --- | --- | --- | --- | --- | --- | --- | --- | --- | --- | --- | --- | --- | --- | --- | --- | --- | --- | --- | --- | --- | --- | --- | --- | --- | --- | --- | --- | --- | --- | --- | --- | --- | --- | --- | --- | --- | --- | --- | --- | --- | --- | --- | --- | --- | --- | --- | --- | --- | --- | --- | --- | --- | --- | --- | --- | --- | --- | --- | --- | --- | --- | --- | --- | --- | --- | --- | --- | --- | --- | --- | --- | --- | --- | --- | --- | --- | --- | --- | --- | --- | --- | --- | --- | --- | --- | --- | --- | --- | --- | --- | --- | --- | --- | --- | --- | --- | --- | --- | --- | --- | --- | --- | --- | --- | --- | --- | --- | --- | --- | --- | --- | --- | --- | --- | --- | --- | --- | --- | --- | --- | --- | --- | --- | --- | --- | --- | --- | --- | --- | --- | --- | --- | --- | --- | --- | --- | --- | --- | --- | --- | --- | --- | --- | --- | --- | --- | --- | --- | --- | --- | --- | --- | --- | --- | --- | --- | --- | --- | --- | --- | --- | --- | --- | --- | --- | --- | --- | --- | --- | --- | --- | --- | --- | --- | --- | --- | --- | --- | --- | --- | --- | --- | --- | --- | --- | --- | --- | --- | --- | --- | --- | --- | --- | --- | --- | --- | --- | --- | --- | --- | --- | --- | --- | --- | --- | --- | --- | --- | --- | --- | --- | --- | --- | --- | --- | --- | --- | --- | --- | --- | --- | --- | --- | --- | --- | --- | --- | --- | --- | --- | --- | --- | --- | --- | --- | --- | --- | --- | --- | --- | --- | --- | --- | --- | --- | --- | --- | --- | --- | --- | --- | --- | --- | --- | --- | --- | --- | --- | --- | --- | --- | --- | --- | --- | --- | --- | --- | --- | --- | --- | --- | --- | --- | --- | --- | --- | --- | --- | --- | --- | --- | --- | --- | --- | --- | --- | --- | --- | --- | --- | --- | --- | --- | --- | --- | --- | --- |

1. Supplementary Table 2: Univariate Cox analysis of inflammation-related genes in PAAD

| id | HR | HR.95L | HR.95H | pvalue |
| --- | --- | --- | --- | --- |
| SYK | 1.3118816 | 1.01338386 | 1.69830348 | 0.03931276 |
| NOD2 | 1.35207102 | 1.01322287 | 1.80423883 | 0.04044195 |
| CXCL8 | 1.16881358 | 1.03202707 | 1.32373 | 0.01403397 |
| HLA-B | 1.4300972 | 1.0802431 | 1.89325719 | 0.01244791 |
| PTGS2 | 1.15228926 | 1.0041753 | 1.32224975 | 0.043454 |
| PRTN3 | 0.44615366 | 0.21250754 | 0.93668717 | 0.03294078 |
| IL1RN | 1.32340374 | 1.13442512 | 1.54386343 | 0.00036479 |
| IFNG | 1.56107951 | 1.07003262 | 2.2774719 | 0.02082028 |
| STAT1 | 1.57681325 | 1.23217516 | 2.01784624 | 0.00029554 |
| IL4 | 0.37685453 | 0.17026293 | 0.83411776 | 0.01606629 |
| PTPN22 | 1.29802087 | 1.01545843 | 1.65920942 | 0.03730295 |
| ICAM1 | 1.23426479 | 1.01833403 | 1.49598219 | 0.03194254 |
| IL18 | 1.57809978 | 1.21439645 | 2.05072973 | 0.00064198 |
| FAS | 1.55906873 | 1.16281648 | 2.09035161 | 0.00299576 |
| MMP9 | 1.14216892 | 1.00139186 | 1.30273661 | 0.04762618 |
| MMP1 | 1.1223206 | 1.03619217 | 1.21560804 | 0.00461613 |
| RELA | 1.6794613 | 1.07029016 | 2.63535101 | 0.02410361 |
| MIF | 0.01529106 | 0.00039114 | 0.59778636 | 0.02541427 |
| VCAM1 | 1.26592016 | 1.05383128 | 1.52069299 | 0.01172213 |
| JAK1 | 1.44865471 | 1.01419431 | 2.06922919 | 0.04160558 |
| PPARG | 1.25029444 | 1.06292642 | 1.47069089 | 0.00700341 |
| ABCB1 | 0.84052578 | 0.71228325 | 0.99185764 | 0.03970973 |
| ALOX5 | 1.2896709 | 1.0526201 | 1.58010571 | 0.01409473 |
| SPP1 | 1.17901217 | 1.04412193 | 1.33132889 | 0.00789673 |
| CASP1 | 1.34848294 | 1.05447871 | 1.7244599 | 0.01718539 |
| CXCL10 | 1.25794237 | 1.10836285 | 1.42770844 | 0.00038108 |
| AGER | 0.70085043 | 0.53413261 | 0.91960557 | 0.01032775 |
| IFNGR1 | 1.6120666 | 1.11478886 | 2.33116676 | 0.01116869 |
| ABCB4 | 0.61892823 | 0.41390405 | 0.92550954 | 0.01943659 |
| IL1R1 | 1.2327599 | 1.01964534 | 1.49041723 | 0.03070589 |
| TLR3 | 1.58950155 | 1.21612545 | 2.07751195 | 0.00069311 |
| F2 | 0.63015863 | 0.41626489 | 0.95395962 | 0.02905375 |
| HFE | 1.9303312 | 1.27117819 | 2.93127948 | 0.00203059 |
| S100A8 | 1.19223463 | 1.02312736 | 1.38929275 | 0.02426402 |
| MMP3 | 1.21640817 | 1.09083537 | 1.35643643 | 0.00042521 |
| EGFR | 1.4388189 | 1.14829534 | 1.80284614 | 0.00156907 |
| MAPK14 | 1.59518157 | 1.02925421 | 2.47227966 | 0.03671245 |
| DNASE1 | 0.60086003 | 0.40154216 | 0.89911549 | 0.01324561 |
| MYD88 | 1.58887062 | 1.1409872 | 2.21256631 | 0.00613184 |
| CD40 | 1.29067966 | 1.01851634 | 1.63556924 | 0.03470311 |
| SERPINE1 | 1.19246181 | 1.01996033 | 1.39413772 | 0.02725362 |
| LCN2 | 1.13491531 | 1.02797467 | 1.25298104 | 0.01219779 |
| TNFSF11 | 1.22136415 | 1.01696726 | 1.4668421 | 0.03235308 |
| NFE2L2 | 1.60910444 | 1.08482338 | 2.38676371 | 0.01804427 |
| IFIH1 | 1.52431988 | 1.15845723 | 2.00572884 | 0.00260943 |
| MMP2 | 1.27879688 | 1.09251833 | 1.49683663 | 0.0022019 |
| DNASE1L3 | 0.7412948 | 0.56656679 | 0.96990857 | 0.02905476 |
| COL7A1 | 1.22659239 | 1.06924653 | 1.4070926 | 0.00354734 |
| MST1 | 0.58314765 | 0.42402425 | 0.80198523 | 0.00090912 |
| MUC5B | 1.1595062 | 1.05148128 | 1.27862917 | 0.00301646 |
| IL4R | 1.34086543 | 1.01439108 | 1.77241315 | 0.03936694 |
| MAPK1 | 1.64113342 | 1.08549793 | 2.4811829 | 0.01882565 |
| PIK3CA | 1.76724584 | 1.21739181 | 2.56545003 | 0.00274986 |
| MUC1 | 1.27450475 | 1.10246783 | 1.47338754 | 0.00104359 |
| TRAF6 | 1.74776441 | 1.02217584 | 2.98840994 | 0.04133969 |
| FERMT1 | 1.51841285 | 1.22615426 | 1.88033239 | 0.00012858 |
| FCGR2B | 1.28403655 | 1.02301644 | 1.6116553 | 0.03106639 |
| NCF2 | 1.22952428 | 1.0250034 | 1.47485361 | 0.02601314 |
| IL1RAPL2 | 1.87105176 | 1.22966533 | 2.84698169 | 0.00344127 |
| ANXA1 | 1.34050843 | 1.11386437 | 1.61326899 | 0.00192805 |
| F2RL1 | 1.30013595 | 1.07203601 | 1.57676932 | 0.00766017 |
| SLC22A4 | 0.70300231 | 0.50292606 | 0.98267378 | 0.0391842 |
| TYK2 | 0.6589642 | 0.45954365 | 0.944924 | 0.02332812 |
| CD80 | 1.54391584 | 1.0945286 | 2.17781074 | 0.01333882 |
| CXCL9 | 1.18173637 | 1.06051618 | 1.31681239 | 0.0024946 |
| TLR1 | 1.39822673 | 1.08882279 | 1.79555205 | 0.00861855 |
| IRF1 | 1.63783882 | 1.1134602 | 2.40917098 | 0.01221736 |
| VDR | 1.58207017 | 1.18712581 | 2.10840839 | 0.00174456 |
| INAVA | 1.34894 | 1.11954361 | 1.62534009 | 0.00164773 |
| ATG16L1 | 1.76100585 | 1.16110846 | 2.67084573 | 0.0077476 |
| TAB2 | 1.80924451 | 1.18769965 | 2.75605511 | 0.00576243 |
| CTNNB1 | 1.74742192 | 1.19297909 | 2.55954477 | 0.00415626 |
| PSMB8 | 2.03220042 | 1.38445097 | 2.98301537 | 0.00029331 |
| MUC5AC | 1.1268151 | 1.03593658 | 1.22566603 | 0.00538788 |
| CASP8 | 1.6055732 | 1.15537377 | 2.23119596 | 0.00479942 |
| SLPI | 1.14898679 | 1.00166867 | 1.3179714 | 0.04728043 |
| UBAC2 | 1.94867591 | 1.09110129 | 3.48027984 | 0.02415774 |
| GZMB | 1.26767781 | 1.05204916 | 1.52750183 | 0.01265438 |
| LAMC2 | 1.38075229 | 1.17204628 | 1.62662251 | 0.00011403 |
| NAMPT | 1.48440034 | 1.12350514 | 1.96122323 | 0.00544688 |
| TNFRSF11B | 1.17486868 | 1.03104052 | 1.3387606 | 0.01557352 |
| SRC | 1.4933292 | 1.078773 | 2.06719311 | 0.01565005 |
| TNFSF4 | 1.31875 | 1.04160542 | 1.66963566 | 0.02152694 |
| EPX | 0.09620451 | 0.0256243 | 0.36119254 | 0.00052304 |
| SERPINB1 | 1.64512606 | 1.25142408 | 2.16268794 | 0.00036107 |
| PTGDR2 | 0.71296356 | 0.55175781 | 0.9212684 | 0.00968133 |
| PYCARD | 1.34573295 | 1.03648724 | 1.74724501 | 0.02581538 |
| GJB2 | 1.28931232 | 1.11483841 | 1.49109165 | 0.000614 |
| MMEL1 | 1.33770148 | 1.03970251 | 1.72111276 | 0.0236504 |
| SREBF1 | 0.66524833 | 0.49468258 | 0.89462488 | 0.00700376 |
| CASP10 | 1.51758497 | 1.09440949 | 2.10438976 | 0.01238961 |
| CD274 | 1.50555473 | 1.0915221 | 2.07663687 | 0.01264227 |
| REL | 1.51933983 | 1.07485484 | 2.14763281 | 0.01784771 |
| HSPD1 | 1.64024582 | 1.15596696 | 2.32740766 | 0.00557458 |
| ADAM17 | 1.59059466 | 1.09337532 | 2.31392763 | 0.01523523 |
| CAPN5 | 1.32025812 | 1.09224355 | 1.59587256 | 0.00407752 |
| TNFSF13B | 1.30214896 | 1.05180075 | 1.61208472 | 0.01536882 |
| EGF | 1.27867762 | 1.00515481 | 1.62663149 | 0.04530267 |
| CD44 | 1.60475879 | 1.21538618 | 2.11887448 | 0.00085106 |
| TGFB2 | 1.19867477 | 1.01405919 | 1.41690072 | 0.03370631 |
| LTB4R | 0.70321186 | 0.53347287 | 0.92695796 | 0.01248642 |
| MMP13 | 1.16067702 | 1.00881027 | 1.33540586 | 0.03729113 |
| LAMA3 | 1.47057382 | 1.23247175 | 1.75467499 | 1.87E-05 |
| CAT | 1.54541946 | 1.10477071 | 2.16182534 | 0.01102937 |
| ITGB4 | 1.37656869 | 1.14874302 | 1.64957813 | 0.00053587 |
| TNFAIP6 | 1.2643693 | 1.06917601 | 1.4951979 | 0.00611076 |
| CD55 | 1.19846107 | 1.0012744 | 1.43448084 | 0.04839882 |
| F3 | 1.26787017 | 1.07684149 | 1.49278681 | 0.00439284 |
| F13A1 | 1.18147778 | 1.01991733 | 1.36863027 | 0.02622699 |
| HSD3B7 | 1.47149476 | 1.04725982 | 2.0675832 | 0.02600904 |
| IL7 | 1.42264269 | 1.05533074 | 1.91779898 | 0.02070168 |
| COL17A1 | 1.25510522 | 1.1256846 | 1.39940541 | 4.27E-05 |
| MME | 1.18431273 | 1.00571279 | 1.39462943 | 0.04253314 |
| NPM1 | 2.80113655 | 1.5120318 | 5.18928634 | 0.0010594 |
| LAMB3 | 1.36228681 | 1.15370005 | 1.60858565 | 0.00026622 |
| GAPDH | 1.3790831 | 1.04989464 | 1.81148671 | 0.0208952 |
| SELPLG | 1.22141047 | 1.00967274 | 1.47755156 | 0.03948788 |
| PLAT | 1.18188807 | 1.03007762 | 1.35607199 | 0.01719824 |
| LGALS3 | 1.54438556 | 1.21476207 | 1.96345179 | 0.00038783 |
| CXCL5 | 1.14893529 | 1.05678313 | 1.24912317 | 0.00113512 |
| KRT7 | 1.35822806 | 1.16386622 | 1.58504769 | 0.00010198 |

1. Supplementary Table 3: Risk score of prognostic models constructed by multifactorial cox regression analysis

AIC = 829.437
Riskscore=(0.0523)*MMP2+(-0.033)*F2RL1+(-0.0796)*COL7A1+(-0.0726)*LAMB3+(-0.0119)*TNFAIP6+(0.0049)*MUC5B+(0.4659)*EGF+(0.0682)*CASP1+(0.1209)*SPP1+(0.062)*PTGS2+(0.0375)*CTNNB1+(0.3838)*IL18+(0.3687)*PSMB8+(0.1612)*LAMA3+(0.1367)*MMP1+(-0.1675)*STAT1+(0.2756)*F13A1+(-0.2316)*PLAT+(0.7102)*UBAC2+(0.183)*LGALS3+(-0.52)*SELPLG+(-0.4045)*IL1RN+(0.2421)*KRT7+(-0.2108)*CXCL10+(-0.892)*IL4R+(0.2808)*LAMC2+(-0.0629)*GAPDH+(0.6929)*PYCARD+(-0.0414)*TGFB2+(0.2229)*ANXA1+(-0.2205)*IRF1+(0.0321)*FCGR2B+(-0.0387)*CD55+(-0.2004)*ITGB4+(-0.0144)*FAS+(0.4231)*MUC1+(-0.0896)*TNFRSF11B+(-1.2794)*NCF2+(-0.1221)*PPARG+(0.1749)*CXCL8+(-0.0541)*SLPI+(0.3235)*MMEL1+(0.7736)*VDR+(-0.4249)*INAVA+(0.1188)*MYD88+(-0.3491)*GJB2+(-0.2685)*ICAM1+(0.6881)*CXCL9+(-0.5284)*CAPN5+(0.3455)*COL17A1+(0.0935)*SERPINE1+(0.0012)*VCAM1+(0.2337)*FERMT1+(0.1312)*CXCL5+(0.0848)*MUC5AC+(-0.0318)*LCN2+(0.308)*MMP9

1. Supplementary Figure 1: GO analysis of targets of BBR and PAAD/Inflammation based on cellular component (CC), biological process (BP)
